# Cytomegalovirus-Specific IL-10-Producing CD4^+^ T Cells Are Governed by Type-I IFN-Induced IL-27 and Promote Virus Persistence

**DOI:** 10.1371/journal.ppat.1006050

**Published:** 2016-12-07

**Authors:** Mathew Clement, Morgan Marsden, Maria A. Stacey, Juneid Abdul-Karim, Silvia Gimeno Brias, Diana Costa Bento, Martin J. Scurr, Peter Ghazal, Casey T. Weaver, Gianluca Carlesso, Simon Clare, Simon A. Jones, Andrew Godkin, Gareth W. Jones, Ian R. Humphreys

**Affiliations:** 1 Division of Infection & Immunity, Cardiff University, Cardiff, United Kingdom; 2 Division of Infection and Pathway Medicine, University of Edinburgh, Edinburgh, United Kingdom; 3 Department of Pathology, University of Alabama at Birmingham, Birmingham, Alabama, United States of America; 4 Respiratory, Inflammation and Autoimmunity, Research Department, MedImmune LLC, Gaithersburg, MD, United States of America; 5 Wellcome Trust Sanger Institute, Cambridgeshire, United Kingdom; ETH Zurich, SWITZERLAND

## Abstract

CD4^+^ T cells support host defence against herpesviruses and other viral pathogens. We identified that CD4^+^ T cells from systemic and mucosal tissues of hosts infected with the β-herpesviridae human cytomegalovirus (HCMV) or murine cytomegalovirus (MCMV) express the regulatory cytokine interleukin (IL)-10. IL-10^+^CD4^+^ T cells co-expressed T_H_1-associated transcription factors and chemokine receptors. Mice lacking T cell-derived IL-10 elicited enhanced antiviral T cell responses and restricted MCMV persistence in salivary glands and secretion in saliva. Thus, IL-10^+^CD4^+^ T cells suppress antiviral immune responses against CMV. Expansion of this T-cell population in the periphery was promoted by IL-27 whereas mucosal IL-10^+^ T cell responses were ICOS-dependent. Infected *Il27rα*-deficient mice with reduced peripheral IL-10^+^CD4^+^ T cell accumulation displayed robust T cell responses and restricted MCMV persistence and shedding. Temporal inhibition experiments revealed that IL-27R signaling during initial infection was required for the suppression of T cell immunity and control of virus shedding during MCMV persistence. IL-27 production was promoted by type-I IFN, suggesting that β-herpesviridae exploit the immune-regulatory properties of this antiviral pathway to establish chronicity. Further, our data reveal that cytokine signaling events during initial infection profoundly influence virus chronicity.

## Author Summary

Viruses including the pathogenic β-herpesvirus human cytomegalovirus (HCMV) can replicate within and disseminate from mucosal tissues. Understanding how to improve antiviral immune responses to restrict virus replication in the mucosa could help counter virus transmission. Studies in the murine cytomegalovirus (MCMV) model have demonstrated the importance of the CD4^+^ T cells in control of mucosal MCMV replication. However, this process is inefficient, allowing virus persistence. Herein, we reveal that production by CD4^+^ T cells of the immune-suppressive soluble protein, or cytokine, interleukin (IL)-10 facilitates virus persistence in mucosal tissue. Mice deficient in T cell-derived IL-10 mounted heightened T cell responses and reduced virus replication in the salivary glands and shedding in the saliva. The cytokine IL-27 induced IL-10-producing CD4^+^ T cells in the periphery whereas a cell surface-expressed protein, ICOS, promoted mucosal IL-10^+^ T cell responses. IL-27 acted in the initial stages of infection to impinge on T cell responses and antiviral control. In turn, IL-27 production in response to viral infection was triggered by type-I interferon, a prototypic antiviral cytokine. Thus, our data suggest that herpesviruses may exploit immune-suppressive properties of this early antiviral cytokine response to facilitate persistence within and shedding from mucosal tissue.

## Introduction

Human cytomegalovirus (HCMV) is a ubiquitous β-herpesvirus that establishes lifelong infection. Infectious virus is usually acquired by horizontal transmission via mucosal secretions and urine. HCMV infection is typically asymptomatic in healthy individuals. However in the immunocompromised such as HIV-infected individuals and patients receiving immune-suppressive drugs, the virus can reactivate with debilitating consequences [1,2]. Further, HCMV is the leading congenital infection in the World, infecting up to 2.5% of live births and causing life-long neurological defects [3].

HCMV employs a range of immune evasion strategies to facilitate persistence. These include down-regulation of MHC and co-stimulatory ligands and modulation of host cytokine production [4,5]. Notably, HCMV encodes its own ortholog of the immune-regulatory cytokine interleukin (IL)-10 [6]. Two isoforms of viral IL-10 exist, both of which suppress immune cell activation *in vitro* [7,8] and induce expression of cellular IL-10 [9,10], suggesting the importance of the immune suppressive functions of IL-10 in HCMV infection *in vivo*. The murine CMV (MCMV) model is well established as a model for HCMV infection *in vivo* due to similar cellular and tissue tropism, and comparable anti-viral immune responses [11]. While MCMV does not encode a vIL-10, cellular IL-10 is induced upon infection of macrophages *in vitro* [12]. Data obtained from the MCMV model has demonstrated an important protective role for cellular IL-10 during acute CMV infection. Myeloid cells and B cells are the predominant sources of IL-10 during initial MCMV infection [13,14]. IL-10 limits virus induced weight loss, pro-inflammatory cytokine production and activation-induced NK cell death [13–15]. Sustained acute MCMV replication in situations of high virus load also induces IL-10 production by NK cells that restricts CD8^+^ T cell-mediated immune pathology [16]. In contrast, production of IL-10 during chronic MCMV infection suppresses viral clearance. Indeed, IL-10-deficient mice exhibit dramatic expansions of virus-specific T cell responses, reduced virus persistence in the salivary glands [14] and fewer viral genome copies in peripheral tissues during chronic/latent infection [17]. Furthermore, persistent MCMV replication in the salivary glands is dramatically restricted by the blockade of IL-10R signaling [18]. These data suggest that although blocking the action of IL-10 during acute CMV infection may be harmful to the host, targeting IL-10-mediated regulation of antiviral T cell responses may impinge on virus chronicity and restrict horizontal virus transmission via mucosal surfaces.

Previous studies have shown that T_H_1 cells can produce IL-10 under certain conditions [19–24]. IL-10 producing T_H_1 cells have been shown to be protective in parasitic infections by limiting infection-related pathology [24–26]. However, in the context of lymphocytic choriomeningitis (LCMV) infection, virus replication is accompanied by the production of IL-10^+^ T cells [27,28], and genetic deletion of IL-10 within T cells (or LysM^+^ cells) reduces virus chronicity [29]. HCMV-encoded latency associated antigens induce CD4^+^/IL-10^+^ responses in healthy donors [30]. MCMV-specific IL-10 production by CD4^+^ T cells has also been described [17,31,32], and CD4^+^ cell-derived IL-10 suppresses control of virus replication and leukocyte accumulation during acute MCMV infection [33]. A substantial proportion of CD4^+^ T cells express IL-10 upon polyclonal stimulation in salivary glands during MCMV persistence [18]. However it is currently unknown how the expansion of these cells is controlled during infection, and whether IL-10 production by T cells impacts on MCMV chronicity.

Type-I IFNs are prototypic antiviral cytokines that exert important control of viral replication. However inappropriate type-I IFN responses promote pathogenesis associated with acute viral infections, and prolonged expression of type-I IFNs in chronic viral infections including human immunodeficiency virus and hepatitis B virus is implicated in driving immune pathology and antagonizing antiviral T cell responses (reviewed in [34]). Further, in the LCMV model of infection, blockade of type-I IFN receptor signaling in certain situations may enhance CD4^+^ T cell immunity and control of virus chronicity, an outcome associated with reduced development of immune-suppressive DCs [35,36]. Although type-I IFN is critical for early control of CMV replication [37], the role that type-I has in shaping adaptive immunity during CMV infection in incompletely understood.

The IL-12 family member IL-27 exhibits a broad spectrum of functions, including the regulation of infection-induced pathologies (reviewered in [38]). Indeed, experiments in murine influenza infection demonstrated that IL-27 restricts virus-induced pathology associated with exuberant neutrophil, T_H_1 and T_H_17 responses [39]. IL-27 induces IL-10 expression by CD8^+^ T cells [40–42], and IL-27-dependent control of influenza-induced inflammation is partially dependent upon IL-10 [39]. However, IL-27 impairs control of acute mouse hepatitis replication and associated pathology following infection of the CNS and this phenotype is associated with reduced accumulation of IL-10^+^ CD4^+^ T cells [43]. Paradoxically, IL-27 exerts cell intrinsic positive regulation of antiviral CD4^+^ T cell responses during chronic LCMV infection, promoting control of virus replication [44]. Moreover, IL-27 inhibits replication of both HIV and HCV via the induction of interferon-stimulated genes [45,46], suggesting that IL-27 is beneficial to the host during chronic viral infections.

Herein we investigated the role that IL-10^+^CD4^+^ T cells play in regulation of MCMV specific T cell immunity. We identified that T cell-derived IL-10 suppressed T cell response during persistent infection and subsequently promoted chronic virus replication. We revealed that an axis involving type-I IFN and IL-27 promotes the generation of peripheral IL-10-producing T cells that suppress antiviral immunity, suggesting that CMV exploits this immune-regulatory pathway induced early in infection to ensure viral persistence within mucosal tissue.

## Results

### Virus-specific CD4^+^ T cells produce IL-10 during MCMV infection in peripheral and mucosal tissue

**Fig 1:**
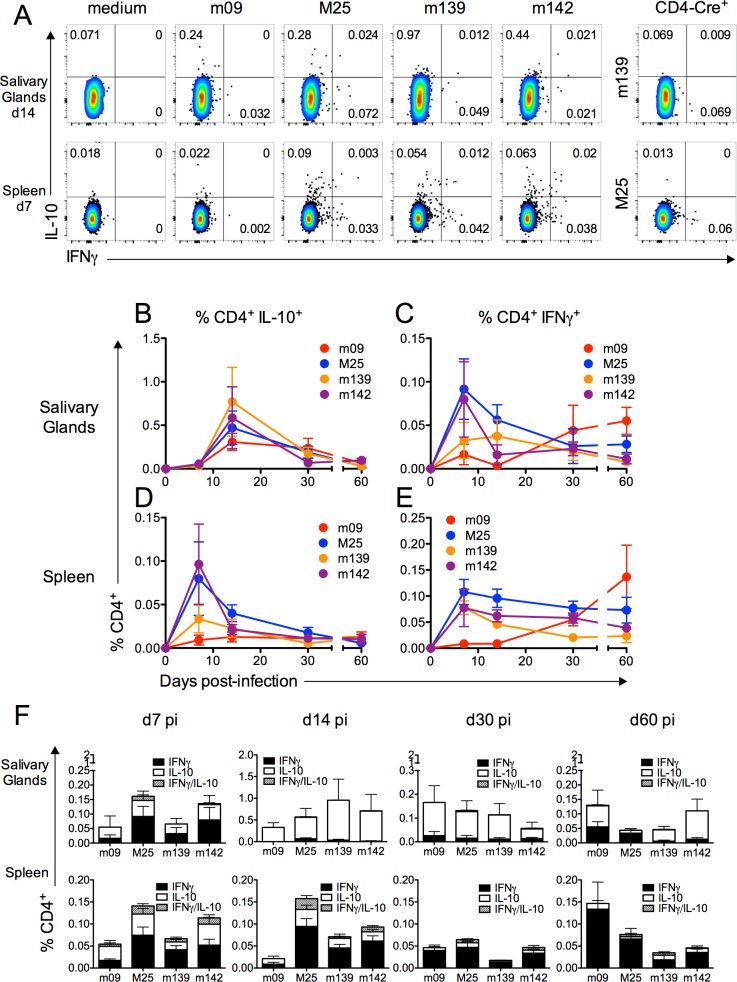
Virus-specific CD4^+^ T-cells produce IL-10 during MCMV infection. CD4-Cre^-^IL-10^flox/flox^ (Cre^-^) mice were infected with MCMV and at day 7, 14, 30 and 60 pi leukocytes from the salivary glands (A-C &F) and spleen (A, &D-F) were isolated. (A) Representative bivariant FACS plots of IL-10 versus IFNγ expression by viable (aqua live/dead^-^) salivary gland (top) and splenic (bottom) CD4^+^CD3^+^ cells. CD4^+^/IFNγ^+^ and CD4^+^/IL-10^+^ responses were measured after 6-hour stimulation with m09, M25, m139 and m142 MHCII MCMV antigens. Data is shown as mean ± SEM percent IL-10^+^ (B&D) and percent + SEM IFNγ^+^ (C&E) CD4^+^ cells and (F) IL-10^+^, IFNγ^+^ and double positive cells over time. Data represents 8–16 mice per group and is representative of 2–3 experiments per time-point.

We previously demonstrated that CD4^+^ but not CD8^+^ T cells produce IL-10 during MCMV infection of the salivary glands, and that IL-10 promotes MCMV persistence [18]. Therefore we sought to assess virus-specific CD4^+^ T cell production of IL-10 over time. Using a panel of MHC class II-restricted peptides identified to induce CD4 T cell responses during acute and persistent MCMV infection [32], we first defined the antigen hierarchy of CD4^+^/IL-10^+^ production in both the mucosa and periphery during MCMV infection using CD4-Cre^-^IL-10^flox/flox^ (Cre^-^) mice, and mice lacking T cell derived IL-10 (CD4-Cre^+^IL-10^flox/flox^, Cre^+^) as controls for IL-10 staining. In accordance with data derived from polyclonally-stimulated CD4^+^ T cells [18], high frequencies of virus-specific IL-10^+^CD4^+^ T cells were observed in salivary glands after MCMV infection (Fig 1A and 1B), peaking at d14 post-infection (pi). Frequencies of salivary gland IL-10^+^CD4^+^ T cells were substantially higher than CD4^+^ T cells producing IFNγ (Fig 1C) suggesting that, at this time-point, IL-10^+^ T cells were the dominant MCMV-specific T cell response. We also detected virus-specific CD4^+^/IL-10^+^ T cells in the periphery (Fig 1A and 1D), highlighting that IL-10 production was not restricted to mucosal CD4^+^ T cells [14,32]. The peripheral IL-10^+^CD4^+^ T cell response reactive to M25, m142 and, to a lesser extent, m139, peaked during acute infection d7 pi (Fig 1D) although, unlike salivary gland responses, were not present at higher frequencies than IFNγ^+^ cells (Fig 1E). Splenic IL-10^+^ CD4^+^ T cell frequencies also contracted rapidly, remaining at low but significant levels during the remaining course of infection.

### MCMV-specific CD4^+^/IL-10^+^ cells express T-Bet

MCMV-induced IL-10^+^CD4^+^ T cells do not express T_H_2-associated cytokines or the regulatory T cell-associated transcription factor FoxP3 [18]. IL-10 is expressed by numerous T helper subsets, including T_H_1 cells [24,25]. Interestingly, although a small CD4^+^IFNγ^+^IL-10^+^ response was detected in the spleen after stimulation with peptide for 6 hours, few co-producing cells were detected in the salivary glands upon stimulation with cognate peptide (Fig 1A and 1F), whereas MCMV-induced IL-10^+^CD4^+^ T cells in the spleen and salivary glands can co-express IFNγ when poly-clonally stimulated with PMA and ionomycin.

**Fig 2:**
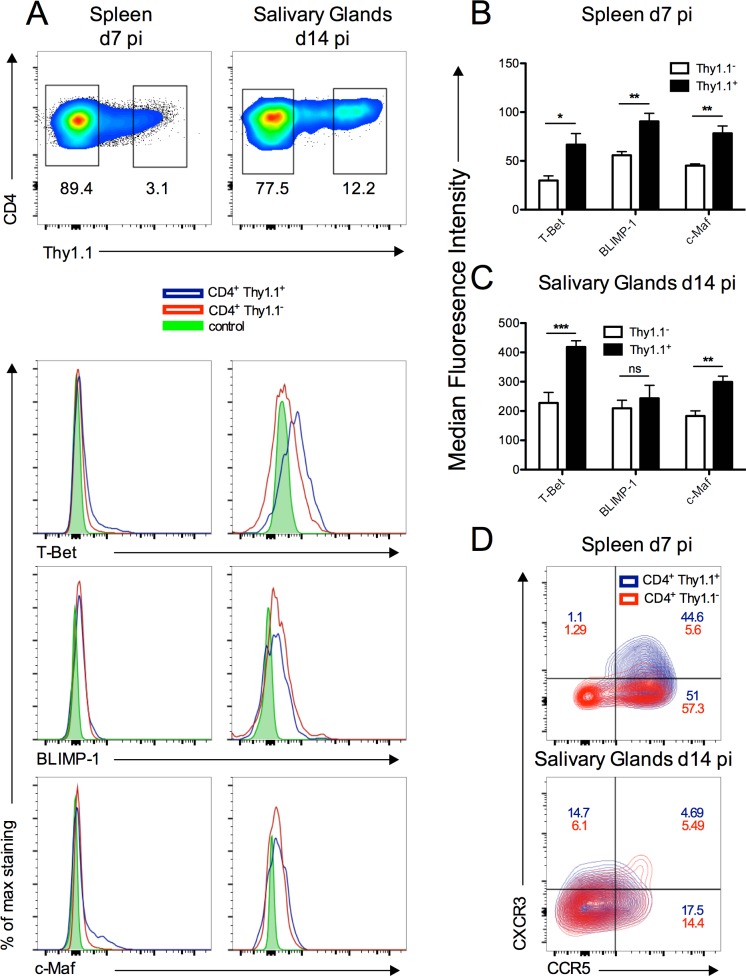
Transcription factor profiling of CD4^+^/IL-10^+^ producing T cells. 10-BiT reporter mice were infected with MCMV and at day 7 and d14 pi spleen and salivary glands were isolated. (A) Representative bivariant FACS plot of viable (aqua live/dead^-^) Thy1.1 expression by CD4^+^CD3^+^ cells (top) and representative histograms of T-Bet, BLIMP-1 and c-maf expression or (D) contour FACS plot of CXCR3/CCR5 expression by Thy1.1^-^ or Thy1.1^+^ CD4^+^CD3^+^ cells. Control = fluorescent minus one-stained Thy1.1^+^ samples from each time-point. (B&C) Intensity of transcription factor staining by CD4^+^CD3^+^ cells in the spleen (B) and salivary glands (C) is shown as mean + SEM of 5–6 mice/group and is representative of 2 separate experiments.

To further investigate the phenotype of IL-10^+^CD4^+^ T cells, we performed transcription factor profiling of these cells using 10-BiT reporter mice that express the surface marker CD90.1 (Thy1.1) under control of the IL-10 promoter without impacting on endogenous IL-10 production [47]. We studied CD4^+^IL-10^+^ cells in the spleen and salivary glands at peak responsiveness (d7 and d14, respectively). In accordance with the ability to express IFNγ in response to polyclonal stimulation, significant expression of the T_H_1-associated transcription factor T-Bet was observed in salivary gland and, to a lesser extent, splenic Thy1.1^+^ CD4^+^ T cells (Fig 2A–2C). In contrast, Thy1.1^+^ CD4^+^ T cells did not express the T_H_17-associated transcription factor, aryl hydrocarbon receptor, nor did they co-express Tr1 cell markers CD49b and LAG-3 (S1 Fig). Instead, splenic Thy1.1^+^ CD4^+^ T cells co-expressed the T_H_1-associated chemokine receptors CXCR3 and CCR5 (Fig 2D). Although Thy1.1^+^ and Thy1.1^-^ salivary gland CD4^+^ T cells expressed low levels of CXCR3 and CCR5 14 days pi (Fig 2D), high expression of their cognate chemokines during salivary gland MCMV infection [48] is consistent with the hypothesis that these receptors are internalized upon binding to their chemokine ligands within infected tissue.

To further explore the transcription control of IL-10 production by CD4^+^IL-10^+^ cells, we examined the expression of Blimp-1 and c-Maf [49]. Significant expression of both transcription factors was detected in Thy.1.1^+^ cells from the spleen and salivary glands of infected mice (Fig 2A–2C). Furthermore, with the exception of Blimp-1 expression by salivary gland CD4^+^ T cells, increased expression of T-Bet, Blimp-1 and c-Maf by Thy1.1^+^ was measured as compared to Thy1.1^-^ cells (Fig 2A–2C). Overall, the combination of transcription factor and chemokine receptor expression of IL-10^+^CD4^+^ T cells induced in response to MCMV indicated that these cells originated from a T_H_1 lineage.

### HCMV specific T-cells in peripheral blood and mucosal tissue secrete IL-10

**Fig 3:**
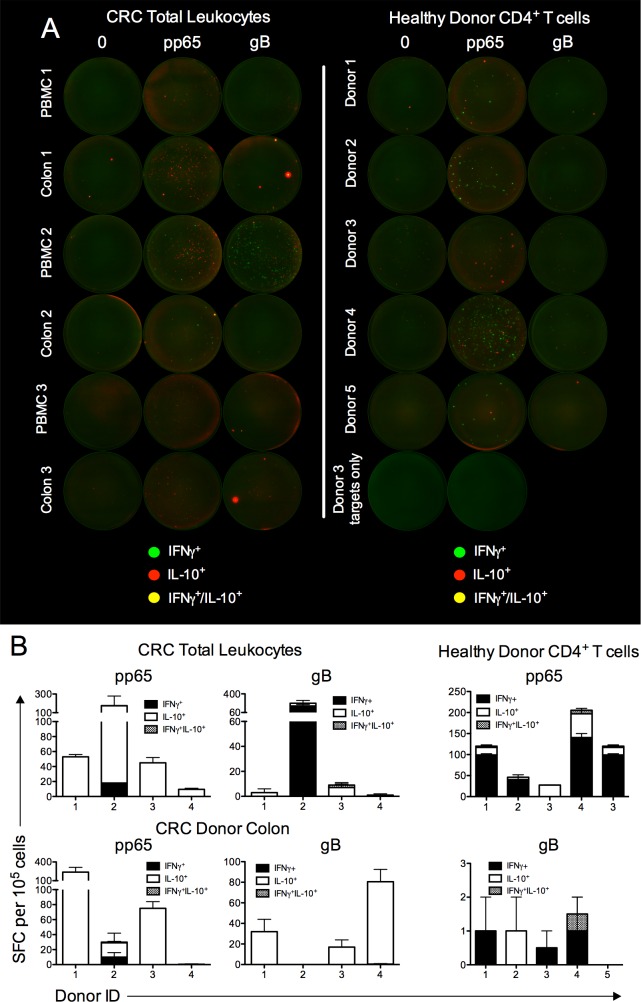
HCMV specific T-cells in peripheral blood and mucosal tissue secrete IL-10. (A) Total leukocytes from colon and peripheral blood (left) or peripheral blood CD4^+^ T-cells isolated by negative selection (right) were isolated from colorectal patients undergoing surgery (left) or HCMV sero-positive healthy donors (right) were stimulated with γ-irradiated autologous targets that were pre-pulsed with vehicle control, pp65 or gB peptide pool. γ-irradiated autologous targets pre-pulsed with vehicle control or pp65 is also shown from healthy CMV sero-positive individuals. After 16hrs, cytokine expression was analyzed using a human IFNγ/IL-10 fluorospot kit where green spots represent IFNγ^+^ cells, red spots represent IL-10^+^ cells and yellow spots indicate dual IFNγ^+^/IL-10^+^ cells. (B) Peptide-specific spot forming cell (SFC) were calculated after subtraction of SFCs in medium-stimulated wells. Data is expressed as mean + SEM of duplicate values. Individual donors are shown.

We next asked whether HCMV-specific T cells reactive to common HCMV antigens would also produce IL-10. Healthy colon and peripheral blood was obtained from colorectal cancer (CRC) patients undergoing colonic resection and we assessed glycoprotein B (gB, UL55) and tegument (pp65, UL83) specific IL-10/IFNγ production using fluorospot. As compared to the low baseline IL-10/IFNγ production by medium-stimulated T cells (Fig 3A), notable albeit variable frequencies of pp65 and gB specific cytokine production were measured in blood (Fig 3B). Interestingly, IL-10 production dominated pp65- and gB-specific responses in 3 of 4 and 2 of 4 patients, respectively (Fig 3B). Importantly, we also detected HCMV-specific cytokine production in leukocytes isolated from the colon (Fig 3A and 3B), demonstrating the presence of HCMV-specific IL-10-producing T cells in human mucosal tissues. To confirm HCMV-specific IL-10 production was derived from CD4^+^ T cells, larger volumes (50mls) of blood were drawn from healthy donors, enabling the purification of CD4^+^ T cells prior to peptide stimulation. Again, both CD4^+^/IFNγ^+^ and CD4^+^/IL-10^+^ cells were observed following stimulation with pp65 and, to a far lesser extent, gB peptide pools (Fig 3A and 3B). As observed in MCMV infection (Fig 1A and 1F), very few virus-specific cells co-produced IL-10 and IFNγ (Fig 3A and 3B). Thus, overall, these data demonstrate the presence of HCMV-specific IL-10^+^ T cells in mucosal tissue and peripheral blood.

### T cell derived IL-10 restricts MCMV-specific T cell immunity

**Fig 4:**
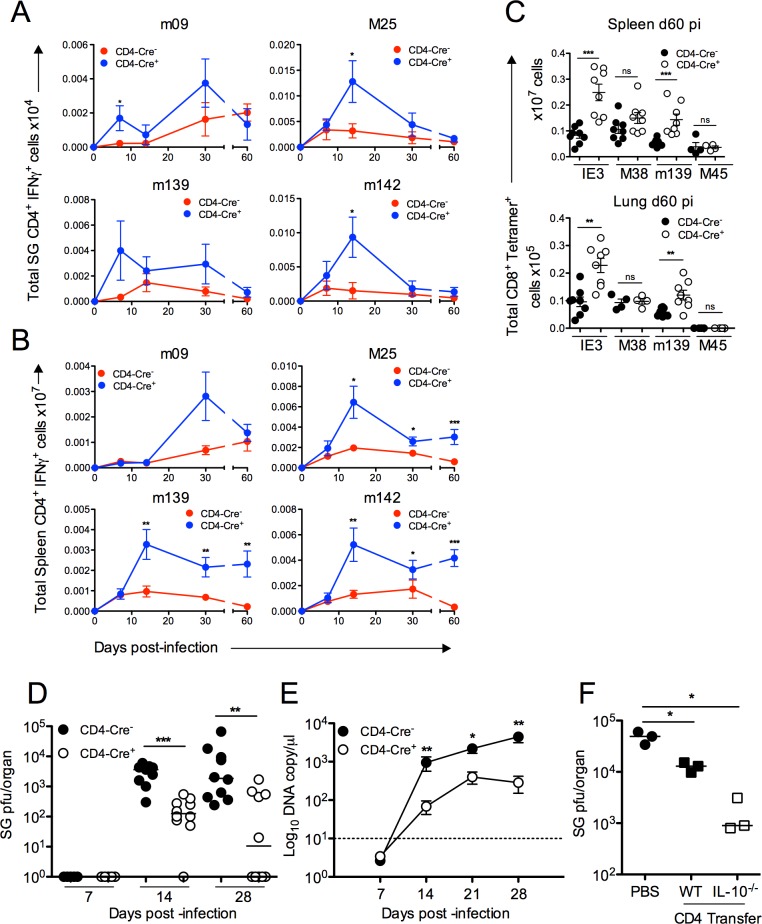
T cell-derived IL-10 impairs anti-MCMV T cell immunity and promotes virus persistence. CD4-Cre^-^IL-10^flox/flox^(Cre^-^) and CD4-Cre^+^IL-10^flox/flox^ (Cre^+^) mice were infected with MCMV and at day 7, 14, 30 and 60 pi peptide-specific CD4^+^ IFNγ^+^ responses in the salivary glands (A) and spleen (B) were measured. Data is shown as mean ± SEM of cell numbers with mean of 8–17 mice per group. (C) Splenic (top) and pulmonary (bottom) virus-specific CD8^+^ T cells were quantified with tetramers refolded around peptides from MCMV antigens IE3, M38, m139 and M45. Total CD8^+^/CD3^+^ Tetramer^+^ cells are plotted with 4–8 mice per group representative of 2 experiments. (D) Replicating virus in salivary gland homogenates at day 7, 14 and 28 pi were measured by plaque assay. Data is shown as individual mice + median and represents 2–3 experiments. (E) Viral genomes were measured in saliva by qPCR day 7, 14, 21 and 28 pi. Data is shown as mean ± SEM from 5 mice/group from 3 replicative experiments. (F) Replicating virus in salivary gland homogenates d14 pi in *rag1* ^-/-^ mice following transfer of WT or *IL-10* ^-/-^ CD4^+^ T cells. Data is shown as individual mice + median and represents two separate experiments.

We sought to investigate the impact of IL-10 production by CD4^+^ T cells on CMV-specific immunity *in vivo*. We utilized CD4-Cre^+^IL-10^flox/flox^ (Cre^+^) mice in which CD4^+^ cells do not produce IL-10. We detected no compensatory IL-10 production by other leukocytes in response to MCMV in these mice (S2 Fig). As compared to CD4-Cre^-^IL-10^flox/flox^ (Cre^-^) control mice, Cre^+^ mice mounted elevated CD4^+^/IFNγ^+^ responses in the salivary glands to multiple MCMV antigens throughout the course of infection (Fig 4A), most notably M25- and m142-specific cells at d14 pi when peak IL-10^+^ responses were observed in Cre^-^ mice (Fig 1B). The absence of CD4^+^ cell-derived IL-10 also consistently increased the accumulation of peripheral virus-specific IFNγ^+^ CD4^+^ T cells during the persistent phase of infection from d14 pi (Fig 4B), supporting the hypothesis that initial production of IL-10 by T cells in the periphery impinged on later T_H_1 cell accumulation.

It has been shown that IL-10 deficiency leads to an increased splenic DC accumulation and expression of co-stimulatory ligands during acute infection [14]. Cre^+^ mice exhibited no notable increased accumulation of splenic DCs or salivary gland myeloid cells either d7 or d14 pi (S3 Fig). However, we observed an increased frequency of myeloid cells expressing co-stimulatory ligands previously demonstrated to enhance MCMV-specific T cell responses during virus persistence [50–52], in the spleens (d7 and d14 pi) and salivary glands (d7 and d14 pi) of Cre^+^ mice (S3 Fig). Although we report some inter-experiment variability regarding the impact of T cell-derived IL-10 on myeloid cell CD86 expression, collectively these data are consistent with a role for T cell-derived IL-10 in modulating myeloid cell function. Furthermore, in accordance with elevated CD4^+^ T cell responses in Cre^+^ mice (Fig 4A and 4B), we observed an increased accumulation of MCMV-specific CD8^+^ T-cells in peripheral tissues (Fig 4C). As observed in studies of IL-10^-/-^ mice [17], T cell-derived IL-10 preferentially suppressed CD8^+^ T cell responses reactive to IE3 (Fig 4C), consistent with observation that IE3-specific CD8^+^ T cell inflation is particularly dependent upon CD4^+^ T cell help [53].

### The absence of IL-10 production by CD4^+^ T cells controls viral replication without inducing autoimmunity

Unlike experiments performed in BALB/c mice [33], we detect no significant virus replication in the salivary glands during acute infection of C57BL/6 mice (Fig 4D). Reduced persistent virus replication is observed in salivary glands of *Il-10* ^-/-^ mice [14] and in WT C57BL/6 mice in which IL-10R signaling is antagonized [18]. Associated with elevated CD4^+^ T cell responses d14 pi in Cre^+^ mice, fewer replicating virions were detectable in salivary glands as compared to controls. Furthermore, 5 out of 10 Cre^+^ mice had cleared replicating MCMV by d30 pi whereas MCMV replication was detectable in all Cre^-^ mice (Fig 4D). Importantly, reduced virus load in Cre^+^ mice was accompanied by decreased shedding of virus in the saliva throughout the course of infection (Fig 4E). Furthermore, improved control of virus replication in Cre^+^ mice was not accompanied by virus-induced Sjögrens Syndrome-like disease, as demonstrated by comparable salivary gland accumulation of TRAILR-expressing CD4^+^ T cells and serum Sjögren Syndrome Antigen (SSA)-specific IgG (S4 Fig), both of which are implicated in this disease [54].

One possible caveat of studies using CD4-cre mice is that CD8^+^ T cells (that express CD4 during thymic development) and CD4^+^ dendritic cells also lack IL-10 expression. To prove that IL-10 derived from CD4^+^ T-cells facilitated MCMV persistence, we negatively selected CD4^+^ T-cell from WT or *Il-10* ^-/-^ mice and adoptively transferred cells into *rag1* ^-/-^ mice. Whereas transfer of WT CD4^+^ T cells reduced MCMV titers by 1 log 14d pi, *Il-10* ^-/-^ T cells reduced virus load by 2 logs (Fig 4F), comparable to virus load in Cre^+^ mice at this time-point (Fig 4D). Thus, two independent approaches supported the conclusion that CD4^+^ T cell derived IL-10 facilitate MCMV persistence.

### MCMV-induced IL-27 is secreted during acute infection and promotes the development of splenic IL-10^+^ CD4^+^ T cells

**Fig 5:**
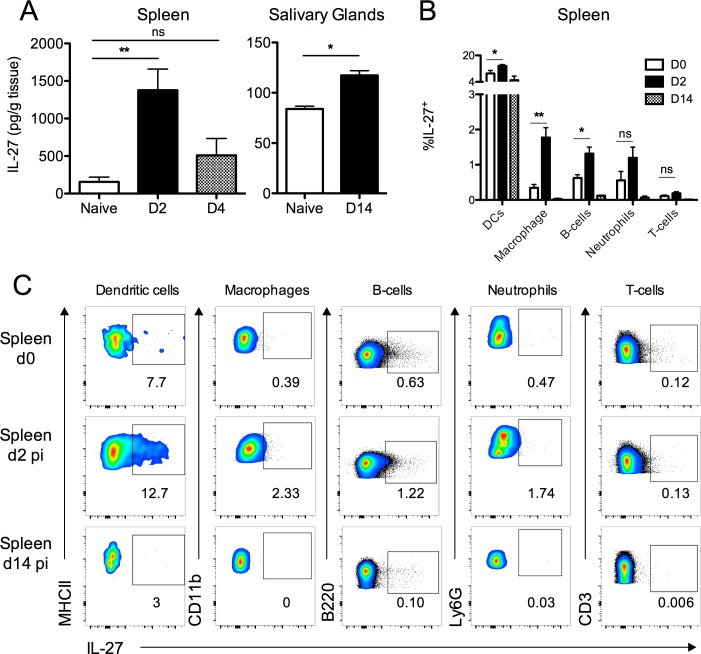
MCMV infection induces myeloid cell expression of IL-27 during acute infection. Spleen and salivary glands were isolated from naïve Cre^-^ mice or after 2 and 4 days of infection. (A) IL-27 release was measured in tissue homogenate supernatants by ELISA. (B&C) IL-27p28 expression by DCs (MHCII^+^/CD11c^+^/Ly6C^+^), Macrophages (CD11b^+^/F480^+^/Ly6C^+^), B-cells (B220^+^/Siglec-H^-^), Neutrophils (Ly6G^+^) and T-cells (CD3^+^) was assessed by intracellular cytokine staining and is shown as mean + SEM of %IL-27 release or by representative concatenated bivariate FACS plots (C). Gating was determined using fluorescent minus one-stained samples from d2-infected mice. Data is shown as 8 mice per group and is representative of at least 2 experiments.

We sought to define the factors promoting the generation of CD4^+^IL-10^+^ T cells. IL-27 is a potent inducer of IL-10 production [38]. We detected a substantial IL-27 p28 production during acute MCMV infection in the spleen that peaked d2 pi (Fig 5A). Elevated IL-27 production in the salivary glands during viral persistence was also detected, albeit much lower than concentrations measured in the spleen (Fig 5A). IL-27 is produced by multiple cells types with predominant expression by myeloid cells [55]. In accordance, we detected a high frequency of IL-27^+^ splenic DCs (Fig 5B and 5C) and, to a lesser extent, macrophages, neutrophils and also B cells (Fig 5B and 5C).

**Fig 6:**
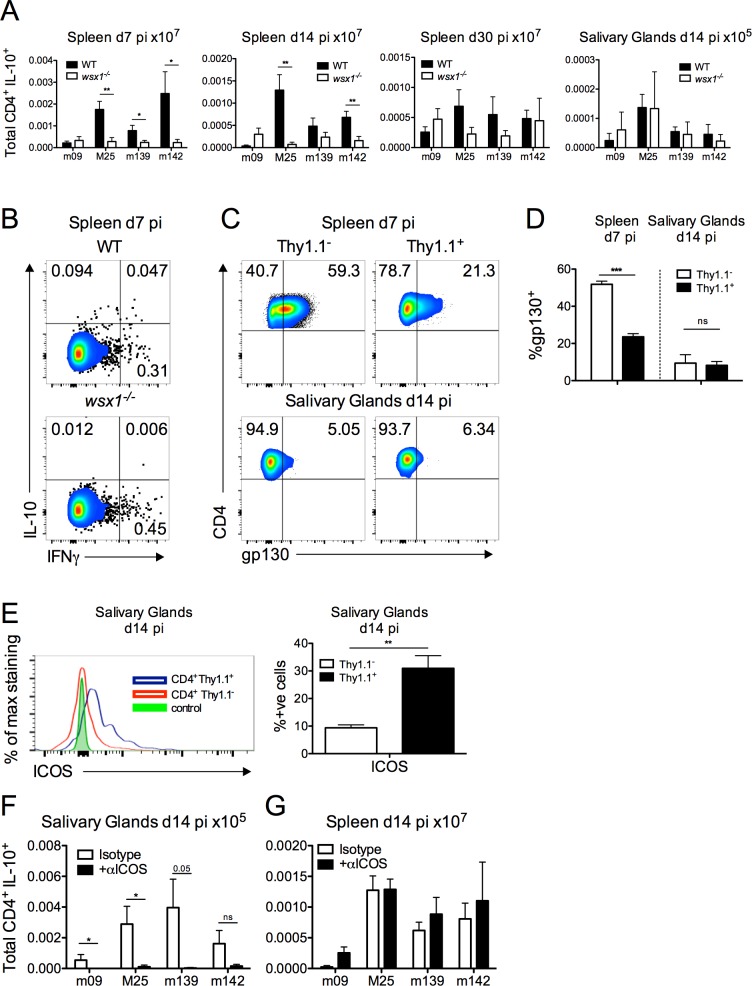
IL-27 promotes splenic CD4^+^IL-10^+^ T cell development whereas ICOS is required for salivary gland CD4^+^IL-10^+^ T cell accumulation. *Il-27r*α^-/-^ or WT (C57BL/6) mice were infected with MCMV and spleen and salivary gland CD4^+^/IL-10^+^ (A) responses were quantified and expressed at mean + SEM of 11 mice/group. (B) Representative bivariant FACS plots of IFNγ versus IL-10 expression by splenic CD4^+^CD3^+^ T cells. (C&D) gp130 and (E) ICOS expression by IL-10^+^ (Thy1.1^+^) and IL-10^-^ (Thy1.1^-^) CD4^+^CD3^+^ T cells was assessed in 10-Bit mice and shown as representative FACS plots (C) and histogram overlays (E) with mean + SEM of 5–6 mice/group (D). Gating was determined using Thy1.1^+^ CD4^+^CD3^+^ cells derived from fluorescent minus one-stained samples from mice infected for 14 days. (F&G) WT (C57BL/6) mice were infected with MCMV and at d6 and d10 pi αICOS or Isotype antibody was added. At d14 pi (F) salivary glands and (G) spleen CD4^+^/IL-10^+^ responses were quantified and expressed as mean + SEM of 6 mice/group.

To establish the link between CMV-induced IL-27 production, and the generation of IL10^+^CD4^+^ T cells, the following experiments were conducted. *Il-27rα* ^-/-^ (*wsx1* ^-/-^) mice were employed which do not respond to IL-27 as a consequence of the loss of the IL-27 receptor complex. Development of splenic IL-10^+^CD4^+^ T cells during acute infection was almost completely abrogated in *Il-27rα* ^-/-^ (*wsx1* ^-/-^) mice, as were the smaller frequencies of IL-10^+^ cells present d14 pi (Fig 6A and 6B). In contrast, development of IL-10^+^CD4^+^ T cells in the salivary glands was comparable in WT and *Il-27rα* ^*-/-*^ mice (Fig 6A). The common IL-27 cytokine receptor glycoprotein 130 (gp130) is down-regulated by virus-specific memory T cells [41] and IL-10^+^ (Thy1.1^+^) and IL-10^-^ (Thy1.1^-^) CD4^+^ T cells in the salivary glands d14 pi expressed less gp130 than splenic T cells isolated d7 pi (Fig 6C and 6D). Importantly, salivary gland IL-10^+^ T cells expressed high levels of the co-stimulatory molecule ICOS (Fig 6E) that has previously been implicated in the development of IL-10-secreting CD4^+^ T cells during viral infection [56]. Monoclonal antibody blockade of ICOS from 6 days pi significantly inhibited the development of salivary gland MCMV-specific IL-10^+^CD4^+^ T cells 14 days pi (Fig 6F). In contrast, splenic IL-10^+^CD4^+^ T cells were unaffected by ICOS blockade (Fig 6G). These data suggest that differential signals are responsible for induction of MCMV-specific IL-10^+^ T cells in different tissues during MCMV infection.

### IL-27 suppresses the development of antiviral T_H_1 immunity and promotes MCMV persistence

**Fig 7:**
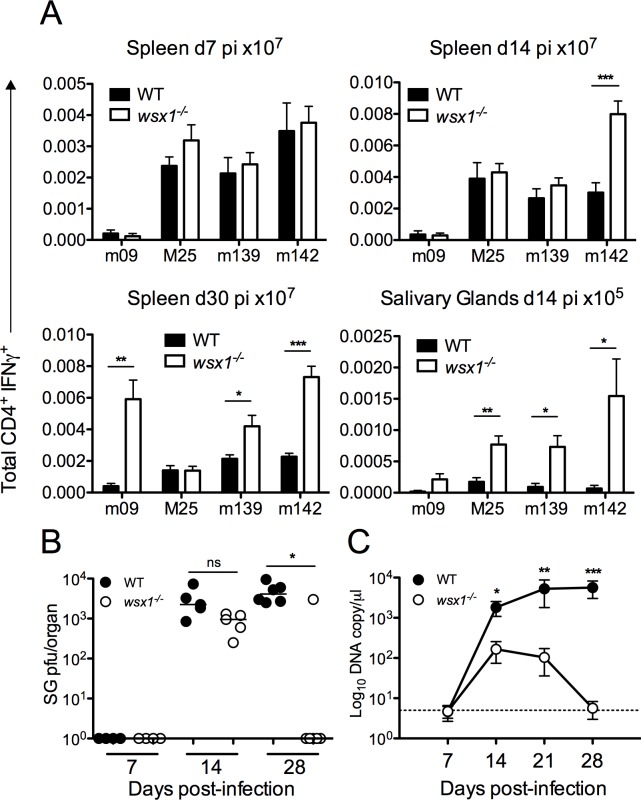
IL-27 promotes CD4^+^IL-10^+^ development and impairs anti-MCMV T_H_1 immunity and control of MCMV persistence. *Il-27r*α^-/-^ or WT (C57BL/6) mice were infected with MCMV and spleen and salivary gland CD4^+^/IFNγ^+^ (A) responses were quantified and expressed as mean + SEM of 11 mice/group. (B) Replicating virus in salivary gland homogenates of *Il-27r*α^-/-^ and WT mice is shown as individual mice + median. Data is representative of 2 experiments. (C) MCMV genomes in saliva were quantified by qPCR. Data is shown as mean ± SEM from 11 mice per group from 2 replicative experiments.

Given the reduced splenic IL-10^+^CD4^+^ T cell accumulation in *Il-27rα* ^*-/-*^ mice, we asked whether antiviral T_H_1 responses were enhanced. IL-27R deficiency did not influence virus-specific T_H_1 responses during acute infection (Fig 7A). However, in accordance with data derived from Cre^+^ mice, *Il-27rα* ^*-/-*^ mice exhibited a significantly increased accumulation of virus-specific T_H_1 cells within the periphery and salivary glands during the persistence phase of infection (Fig 7A). As observed in Cre^+^ mice (Fig 4C), elevated T_H_1 cell were also accompanied by increased virus-specific CD8^+^ T-cell response d30 pi (S5 Fig). In contrast, blockade of ICOS from 6 days pi did not enhance virus-specific IFNγ^+^CD4^+^ T cell responses either in the spleen or salivary glands 14 days pi (S6 Fig), suggesting a dominant inhibitory effect of IL-27-dependent IL-10^+^ T cell responses during the first 14 days of MCMV infection.

Given that IL-27 induction of IL-10 significantly impaired anti-MCMV T cell immunity, our attention focused on whether IL-27 impacted on viral persistence. In the absence of IL-27 signaling, viral load was decreased d14 pi and MCMV replication was absent in salivary glands of most mice by d30 pi (Fig 7B). Furthermore, we detected a significant decrease in virus shedding by *Il-27rα* ^*-/-*^ mice in the saliva, with few viral genomes detected in the saliva by d30 pi (Fig 7C). These data are consistent with the hypothesis that IL-27 induction of IL-10^+^CD4^+^ T cells in the periphery during acute infection contributes to the suppression of T_H_1 immunity and subsequent viral persistence and transmission.

### MCMV-induced IL-27 production during acute infection is dependent upon type-I IFN and promotes virus persistence

**Fig 8:**
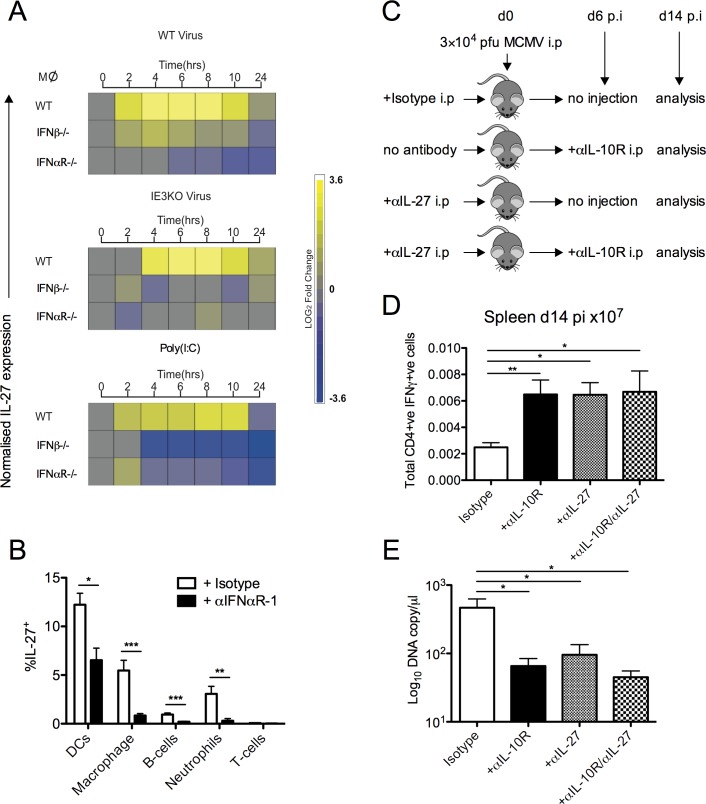
Type-I IFN induces IL-27 expression during MCMV infection. (A) Heat map of IL-27 p28 mRNA expression by WT, IFNβ^-/-^ and IFNαR^-/-^ macrophages following stimulation with WT MCMV (C3X), replication-deficient (ΔIE3) MCMV or poly(I:C). (B) Expression of IL-27 p28 by splenic leukocytes was assessed d2 pi in Cre^-^ mice treated/not with 2 mg anti-IFNαR-1. Mean + SEM of 8 mice/group is shown and data is representative of 2 separate experiments. (C) WT (C57BL/6) mice were infected with MCMV treated with anti-IL-27, Isotype or virus alone. At d6 pi mice were treated /not with anti-IL-10R and at d14 pi spleen CD4^+^/IFNγ^+^ (D) responses were quantified and expressed as mean ± SEM of 4–15 mice/group. (E) MCMV genomes in saliva were quantified by qPCR. Data shown as mean ± SEM from 4–15 mice/ group.

We examined the factors regulating IL-27 production in response to MCMV. Type-I interferon exerts critical control of MCMV infection *in vivo* [57,58]. However, type-I IFN induction of IL-27 during bacterial infection has been described [59]. Hence a paradoxical situation may arise where type-I IFN controls CMV on one hand, but induces IL-27 leading to inferior control of persistent virus replication. We examined whether type-I IFN orchestrated IL-27 production in response to MCMV, using an established *in vitro* MCMV infection system of bone marrow-derived macrophages [60]. Productive infection of WT macrophages induced substantial IL-27 mRNA expression, peaking 6hrs pi (Fig 8A). Similarly, IL-27 expression was also maximally induced 6hrs pi with by a non-productive viral replication, as demonstrated using replication-deficient (ΔIE3) MCMV. As the primary macrophage response to viral infection involves type-I IFN signaling we next directly tested the dependency of IL-27 induced expression in macrophages that are genetically ablated for either IFNβ production or type-I IFN signaling. MCMV infection of either *Ifnβ* ^*-/-*^ or *Ifnαr* ^*-/-*^ macrophages failed to induce IL-27, a result further mirrored by IFN-dependent IL-27 expression upon stimulation with the TLR3 agonist, Poly:(IC) (Fig 8A). Moreover, administration of a blocking anti-IFNαR-1 monoclonal antibody during MCMV infection *in vivo* inhibited IL-27 expression by all cellular subsets examined (Fig 8B). In the case of macrophages, B-cells and neutrophils, IL-27 production was almost completely abrogated by IFNαR-1 blockade (Fig 8B). To examine whether early IFN-induced IL-27 production shaped antiviral responses, we treated MCMV-infected mice with neutralizing anti-IL-27 antibody at the time of infection (Fig 8C). IL-27 neutralization significantly enhanced total virus-specific T_H_1 responses and reduced virus shedding 14 days pi (Fig 8D and 8E) whilst concurrently reducing accumulation of virus-specific IL-10^+^CD4^+^ T cells in the spleen at this time (Pooled m09, M25, m139 and m142-specific CD4^+^IL-10^+^ T cells: Isotype control = 0.00162 × 10^7^ cells versus αIL-27 treated group = 0.000908 × 10^7^ cells). Importantly, IL-10R blockade from the time of IL-27-induced IL-10^+^ T cell responses (day 6 pi) had no additional inhibitory influence on IFNγ^+^ T cell responses and virus shedding (Fig 8D and 8E). Thus, IL-27 production in response to MCMV is dependent upon type-1 IFNR signaling, and this axis acts during the initial days of infection to promote the accumulation of MCMV-specific IL-10^+^CD4^+^ T cells, subsequently promoting virus persistence and shedding from the mucosa.

## Discussion

Herein we reveal that cytokine responses induced upon acute MCMV infection influence antiviral T cell responses and virus replication during pathogen persistence. We identified that IL-27, which was produced by myeloid cells during the initial days of infection, promoted the generation of IL-10^+^CD4^+^ T cells in the spleen. Our data suggest that this IL-27-dependent induction of peripheral IL-10^+^CD4^+^ T cells was sufficient to impinge on T cell mediated control of virus chronicity in the mucosa. IL-27 production was dependent upon type-I IFN, implying that CMV exploits the immune-regulatory actions of this prototypic antiviral cytokine pathway to enable persistence and dissemination.

The dynamics of virus-specific IFNγ versus IL-10 production by CD4^+^ T cells suggested that contraction of the IL-10 response preempts expansion of IFNγ^+^ T cell numbers and subsequent control of virus replication. Preventing IL-10 production by T cells both enhanced and accelerated the generation of virus-specific T_H_1 responses. These data suggest that IL-10^+^ T cells provide a window of opportunity for MCMV to replicate within and shed from the salivary glands. Conversely, these results also imply that inhibiting virus-specific IL-10^+^ T cell development may be therapeutically beneficial. Our data suggest that peripheral IL-10^+^ T cells inhibit the control of virus replication within mucosal tissue independently of mucosal IL-10^+^ T cell populations, highlighting that appropriate modulation of peripheral T cell responses via systemic vaccination approaches may be capable of overcoming inhibitory pathways that act in the mucosa, thus enabling effective control of virus replication within mucosal tissues.

Using a combination of conditional knockout mice and a cell transfer model, we demonstrated an important role for CD4^+^ T cell-derived IL-10 in facilitating MCMV persistence. This result is in apparent contradiction to bone marrow chimera experiments suggesting no role for CD4^+^ cells in IL-10-mediated chronic MCMV replication [14]. There are several explanations for these apparent discrepancies. Firstly, whereas we used salivary gland-propagated Smith strain MCMV in our experiments, Mandaric et al examined tissue culture-propagated virus deficient in m157, a viral ligand for the NK cell activating receptor Ly49H [61]. These viruses likely replicate with different kinetics and, possibly, tropisms that may influence the induction of IL-10 expression by T cells and other cells. Furthermore, the mixed chimera experiments performed by Mandaric et al used IL-10^-/-^ mice as recipients whereas our experiments were performed in mice from IL-10-sufficient backgrounds. Given that the time-point assessed by Mandaric et al (d14 pi) was prior to the maximal impact of CD4-derived IL-10 in our model, such variations in experimental design may influence the impact of CD4^+^ T cell-derived IL-10 on virus persistence.

Importantly however, significant expression of IL-10 by HCMV-specific T cells in peripheral blood and mucosal tissue highlights the potential importance of T cell-derived IL-10 in regulating anti-CMV immunity and viral persistence, as suggested by findings obtained from our model of chronic MCMV infection, and further supported by experiments studying acute MCMV infection [33]. HCMV-specific IL-10 production was detected in response to common lytic antigens pp65 and gB, although T cell responsiveness to gB was low in peripheral blood of all healthy volunteers examined. IL-10 production by CD4^+^ T cells reactive to latency-associated HCMV antigens have also been described [30]. Persistent shedding of HCMV in children is associated with poor T_H_1 responses [62]. Our data derived from murine experiments demonstrates that preventing the generation of HCMV-specific IL-10^+^ CD4^+^ T cells may be beneficial for the improvement of protective T cell immunity following vaccination, particularly in the context of restricting horizontal transmission via mucosal surfaces.

IL-27 promoted the development of splenic but not salivary gland IL-10^+^CD4^+^ T cells. Why IL-27 had no impact on salivary gland CD4^+^IL-10^+^ accumulation is incompletely understood. Salivary glands APCs, which are phenotypically indicative of tissue-resident macrophages [63], are tightly regulated via inhibitory signals such as CD200 expressed by endothelial cells [60]. Thus, restricted activation of this cell population may contribute to the relatively low IL-27 production measured in this organ. Furthermore, the infection phase may also influence the impact of IL-27 on IL-10^+^ T cell development. Memory CD8^+^ T cells generated in influenza infection down-regulate gp130 [41] and CD4^+^ T cells in persistently-infected salivary glands expressed less gp130 expression than splenic CD4^+^ T cells during acute infection. This down-regulation of gp130 correlated with a reduced impact of IL-27R signaling on salivary gland IL-10^+^ CD4^+^ T cell accumulation during virus persistence.

Instead, ICOS co-stimulation was required for the accumulation of IL-10^+^CD4^+^ T cells within the salivary glands, thus demonstrating that differential signals promote IL-10^+^ T cell responses in different anatomical locations of MCMV infection and suggesting that ICOS acts independently of IL-27 in the induction of salivary gland CD4^+^IL-10^+^ T cells. This contrasts *in vitro* findings demonstrating that ICOS acts downstream of IL-27 in promoting IL-10 production by Tr1 cells *in vitro* [64]. However, salivary gland IL-10^+^CD4^+^ T cells in our model were phenotypically distinct from Tr1 cells. Moreover, here we examined the influence of ICOS co-stimulation of T cells during virus persistence rather than during initial T cell activation as studied by Pot et al. Therefore, although the impact of ICOS on CD4^+^IL-10^+^ T cell generation during acute infection was not examined in our study, these data collectively imply that the type of IL-10-producing T cell and the timing of IL-10-inducing signals may influence the relationship between IL-27 and ICOS in the induction of CD4^+^IL-10^+^ T cells. Notably, blockade of ICOS was not accompanied by an elevated virus-specific T_H_1 responses d14 pi despite dramatically reducing salivary gland CD4^+^IL-10^+^ accumulation. This may reflect a possible dual function for ICOS in positive co-stimulation [65] in addition to IL-10 induction. However, our data does not preclude a possible role for ICOS and, possibly, other IL-10-inducing receptors in the suppression of antiviral control during the latter stages of MCMV persistence.

We demonstrate that IL-27 is critical for the induction of peripheral IL-10^+^CD4^+^ T cell responses and subsequent suppression of T_H_1 responses during the persistent phase of infection. Experiments using anti-IL-27 and anti-IL-10R suggest that early IL-27-mediated induction of IL-10^+^ T cells has a long-term impact on antiviral immunity and virus persistence. However, our data does not preclude an IL-10-independent inhibitory function for IL-27 during the later stages of infection. Indeed, antagonizing both IL-10R and IL-27 14 days pi led to an additive, albeit modest, increase in virus-specific IFNγ^+^CD4^+^ T cells by day 30 (S7 Fig). Intriguingly, IL-27 can induce dendritic cell expression of the ATPase CD39 [66]. However, in contrast to *IL-27rα* ^-/-^ mice, mice deficient in the ecto-5’-nucleotidase CD73 that acts downstream of CD39 in the purinergic system have no alteration in MCMV-specific memory T cell accumulation and only a moderate and transient reduction in MCMV replication in the salivary glands [67]. Taken together with data derived from CD4^-Cre^IL-10^flox^ and *IL-27rα* ^-/-^ mice, these results collectively suggest that early induction of IL-10^+^CD4^+^ T cells is the dominant mechanism through which IL-27 impinges on anti-MCMV T cell immunity.

*In vivo* and *in vitro* experiments revealed that the induction of IL-27 during MCMV infection was promoted by type-I IFN. Although the uncontrolled virus replication and associated disease and inflammation observed in mice treated with anti-IFNαR-1 antibody precluded the assessment of the direct impact of IFNαR-1 signaling on both IL-27-driven IL-10^+^ T cell development and virus persistence, our data implies that type-I IFN may impinge on antiviral immunity via the induction of IL-27. Comparable IFN-dependent induction of IL-27 was observed upon *in vitro* stimulation with either MCMV or the TLR3 ligand, poly(I:C). Endosomal TLRs including TLR3 are activated upon acute MCMV infection and induce type-I IFN expression [68,69]. Thus, our data suggest that MCMV may exploit an immune-regulatory aspect of this “antiviral” response to promote persistence within mucosal tissue and subsequently increase the window of opportunity for horizontal transmission via mucosal secretions.

## Materials and Methods

### Mice, infections and treatments

CD4-Cre^-^IL-10^flox/flox^ (Cre^-^)/CD4-Cre^+^IL-10^flox/flox^ (Cre^+^) were generated by Werner Muller (University of Manchester) and were kindly provided by Jean Langhorne (Francis Crick Institute), 10-BiT mice were given to us by Padraic Fallon (Trinity College Dublin) and *Il-27rα* ^*-/-*^ (*wsx1* ^*-/-*^ *)*, *Rag1* ^-/-^ and *Il-10* ^*-/-*^ mice were obtained from The Jackson Laboratory. All mice were bred in-house. C57BL/6 WT mice were purchased from Charles River or Envigo. Mice were infected with MCMV that was prepared by sorbital gradient purification as described previously [70]. Mice were infected with 3 × 10^4^ pfu MCMV intraperitoneally (i.p.). In some experiments, CD4-Cre^-^IL-10^flox/flox^ mice were administered 2 mg αIFNαR-1 (clone MAR1-5A3, BioXcell) or IgG1 Isotype control (clone MOPC-21 BioXcell) at the time of infection, or 200 μg anti-ICOS (clone 7E.17G9 or JmAb51-TM) or IgG control (clone LTF2 or R347-TM), respectively) on days 6 and 10 pi. In other experiments mice were treated with 500 μg anti-IL-27 (clone MM27-7B1, Biolegend), 250 μg anti-IL-10R (clone 1B1.3A BioXcell) and also in combination or Isotype control (clone MOPC-21 BioXcell) on days stated in the legend. In some experiments, CD4^+^ T cells were isolated from spleens of C57BL/6 and IL-10^-/-^ mice by negative separation (Miltenyi Biotec). 5 x 10^6^ cells were then transferred intravenously (i.v.) into *rag1* ^-/-^ mice one week prior to MCMV infection.

### T-cell effector function assays

Leukocytes were extracted from murine spleen, salivary glands and lungs as described previously [13,60]. For CD4^+^ functional responses, isolated leukocytes were stimulated for 2 hours with 3 μg/ml m09, (GYLYIYPSAGNSFDL), M25 (NHLYETPISATAMVI), m139 (TRPYRYPRVCDASLS), and m142 (RSRYLTAAAVTAVLQ) MCMV MHCII peptides (Genscript). Brefeldin A (Sigma) was then added and cells incubated for a further 4 hours. For CD8^+^ functional responses, leukocytes were stimulated with 2 μg/ml m139, IE3, M38, and M45 MCMV MHCI peptides in the presence of anti-mouse CD107a-FITC (Biolegend) with monensin (BD Biosciences) and brefeldin A for 6 hours. Cells were subsequently stained with Zombie Aqua fixable viability dye (Biolegend) or LIVE/DEAD-Aqua (Life-Technologies), stained with anti-CD16/CD32 Fc-block (Biolegend) and then with either anti-CD4 Pacific-Blue or PercP, (clone RM4-5, Biolegend) or with anti-CD8 PercP (clone 53–6.7, Biolegend) and anti-CD262 (TRAILR DR5, clone MD5.1, eBioscience). Cells were fixed, saponin permeabilised and stained for anti-IFNγ FITC or Pacific-Blue (clone XMG1.2, Biolegend) and anti-IL-10 APC (clone JES5-16E3, eBioscience). Data was acquired using a BD FACSCantoII or a BD LSR II fortessa flow cytometer (BD Biosciences) and analysed with FlowJo software (TreeStar).

### Cell phenotyping and IL-27 detection

Direct *ex vivo* intracellular IL-27 production was detected as previously described [71]. Cells were stained with a combination of anti-I-A/I-E (clone M5/114.15.2, Biolegend), anti-CD11c Pe-Cy7 (clone HL3 BD, Pharmingen), anti-Ly6C FITC (clone AL-21 BD Pharmingen), anti-CD11b APC-Cy7 (clone M1/70, Biolegend), anti-F480 Brilliant-Violet 711 (BV711) (clone BM8, Biolegend), anti-CD45R/B220 BV785 (clone RA3-6B2, Biolegend), SiglecH APC (clone 551, Biolegend), anti-Ly6G PerCP-Cy5.5 (clone 1A8, Biolegend), and anti-CD3ε BV605 (clone 145-2C11, Biolegend). Cells were fixed, saponin permeabilised and stained with anti-IL-27 p28 PE (clone MM27-7B1, Biolegend). To determine T cell transcription factor profiles cell were surface-stained with anti-CD4 BV785 (clone RM4-5, Biolegend), anti-CD90/CD90.1 (Thy1.1) BV650 (clone OX-7, Biolegend) anti-CD130 PE (gp130) (clone KGP13, eBioscience), fixed and permeabilised using BD Cytofix/Cytoperm solution (BD Biosciences) and stained with anti-T-Bet BV421 (clone 4B10, Biolegend), anti-BLIMP-1 Alexa-Fluor 647 (AF647) (clone 5E7, Biolegend), and anti-cMaf eFluor 660 (clone sym0F1, eBioscience). All data was acquired using a BD LSRForetssa flow cytometer (BD Biosciences) and analyzed with FlowJo software (TreeStar). For analysis of co-stimulatory ligand expression, cells were surface stained with a combination of anti-CD11c APC-Cy7 (clone N418, Biolegend), anti-I-A/I-E PerCP-Cy5.5 (clone M5/114.15.2, Biolegend) anti-F480 Brilliant-Violet 421 (BV421) (clone BM8, Biolegend), anti-CD80 APC (clone 16-0A1, Biolegend), anti-CD86 Pe-Cy7 (clone GL-1, Biolegend), anti-OX40L APC (clone RM134L, Biolegend), anti-4-1BBL PE (clone TKS-1, Biolegend), anti-CD40 FITC (clone 3/23, Biolegend) and anti-Ly6G PE (clone 1A8, Biolegend), anti-ICOS APC-Cy7 (clone C398.4A, Biolegend), anti-AhR Alexa-Flour 488 (AF488) (clone 4MEJJ eBioscience), anti-CD49b Pe-Cy7 (clone DX5, Biolegend), anti-LAG3 PE (clone C9B7W, Biolegend) anti-NK-1.1 Pe-Cy7 (clone PK136, Biolegend), anti-CD19 FITC (clone 6D5, Biolegend), anti-CXCR3 PerCP-Cy5.5 (clone CXCR3-173 Biolegend), and anti-CCR5 AF488 (clone HM-CCR5, Biolegend). Data was acquired using a BD FACSCantoII flow cytometer (BD Biosciences) and analysed with FlowJo software (TreeStar).

### Tetramer staining

The following MCMV MHCI biotinylated were refolded and kindly provided by the NIH tetramer Core Facility- H-2D(b) M45 985–993 HGIRNASFI, H-2k(b) m139 TVYGFCLL, H-2k(b) M38 316–323 SSPPMFRV, H-2k(b) IE3 RALEYKNL. Biotinylated monomers were tetramerised as described previously [72]. Isolated leukocytes were stained with viability dye and then with 25 μg/ml tetramer conjugated to PE or APC for 15 minutes at 37°C. Cells were subsequently stained with Fc-block and anti-CD8 APC-Cy7 (clone 53–6.7, Biolegend). Data was acquired using a BD FACSCantoII flow cytometer (BD Biosciences) and analysed with FlowJo software (TreeStar).

### Isolation of human T cells from human colon

Peripheral blood and healthy colon specimens were obtained from patients undergoing primary tumor resection for colorectal adenocarcinoma at the University Hospital of Wales, Cardiff. Blood samples were collected on the day of but prior to surgery. The tissue used in this study was autologous colon samples that were cut from a macroscopically normal section of the excised tissue, at least 10 cm from the tumor. Colon tissue was washed in extraction medium consisting of DMEM supplemented with 100 U/ml Penicillin, 100 mg Streptomycin and 2 mM L-Glutamine, 20 μg/ml Gentamincin and 2 μg/ml Fungizone (all Life-Technologies) as described previously [73]. Samples were finely cut with scalpel blades in a petri dish and forced through a 70 μM and subsequently a 40 μM cell strainer. Cells were washed and pelleted by multiple centrifugations and lymphocytes were isolated using Lymphoprep (Axis-Shield, Scotland).

### Fluorospot analysis of IFNγ and IL-10

Peripheral blood mononuclear cells (PBMCs) from healthy HCMV sero-positive healthy volunteers were isolated using Lymphoprep. For target cells, some PBMCs were γ-irradiated with 3000 Rad, plated out @ 1 × 10^5^ cells/well and pulsed with 2 μg/ml of Peptivator CMV pp65 overlapping peptide pool (Miltenyi Biotec), 2 μg/ml PepMix HCMVA UL55 overlapping peptide pool (JPT), or with DMSO vehicle control for 2 hours @ 37°C. CD4^+^ T cells were then isolated from the remaining PBMCs by negative selection (Miltenyi Biotec). Pulsed autologous targets were then transferred to a human IFN^+^/IL-10^+^ Flurospot plate thus removing (IL-10-producing) monocytes that adhered to the plate. 3 × 10^5^ purified CD4^+^ T-cells and 1/100 human anti-CD28/CD49d co-stimulation antibodies (BD Pharmingen) were then added for 18 hours @ 37°C. E:T ratios ranging from 1:3 to 3:1 were tested. After incubation, plates were assayed according to the manufacturer’s instructions (Mabtech) and quantified using a CTL Immunospot Fluorospot Line plate reader (CTL). Cells were maintained in RPMI, 100 U/ml Penicillin, 100 mg Streptomycin, 2 mM L-Glutamine (all Life-Technologies) and 5% human AB serum (Welsh Blood Transfusion Service). Autofluoresence is adjusted using the analysis software and removed from the final SFC count.

For analysis of colon leukocyte cytokine production, PBMCs from the colon donor was used as autologous targets and γ-irradiated in an identical manner as that described previously. 1.5 × 10^5^ cells per well and pulsed with peptide pools or DMSO vehicle control for 2 hours @ 37°C. Cells were transferred to a human IFN^+^/IL-10^+^ Fluorospot plate with the addition of 5 × 10^4^ cells from peripheral blood and Colon processed tissue with the inclusion of 1/100 human anti-CD28/CD49d co-stimulation antibodies for 18 hours @ 37°C. Plates were assayed and measured as described above.

### Antibody and cytokine measurements

Cardiac punctures were performed on CD4-Cre^-^IL-10^flox/flox^, CD4-Cre^+^IL-10^flox/flox^ mice on either naïve mice or after 60 days of MCMV infection and plasma isolated. Plasma was then assayed for anti-Sjögrens Syndrome Antigen IgG by ELISA (Alpha Diagnostics). Samples were analysed according to the manufacturer’s instructions and Total IgG antibody levels present in the plasma was calculated by interpolation from the calibrator curve provided with the kit. Excised tissue from both Spleen and Salivary Gland (approx. 50–100 mg) were weighed, washed and re-suspended in DMEM. Supernatant was assayed in triplicate for IL-27 p28 production by ELISA and performed according to the manufacturer’s instructions (eBioscience).

### Viral load quantification

Infectious virus quantification was determined using plaque assays as previously described [60]. Viral DNA copy number in saliva was determined using qPCR for relative expression of IE1, as described previously [74]. Oral lavage was performed on the sublingual cavity of anaesthetised mice using 20 μl of sterile PBS. 1 μl of sample was used for qPCR and measured using SYBR green (Bio-Rad) using MCMV IE1 forward (5′-AGCCACCAACATTGACCACGCAC-3′) and MCMV IE1 reverse (5′-GCCCCAACCAGGACACACAACTC3′) primers. PCR was performed using an MJ Mini Personal Thermal Cycler (Bio-Rad) using the conditions as described previously [74]. To establish a standard curve, DNA copy/μl was determined from the known concentration and molecular weight of the MCMV pARK25 BAC (a kind gift from Alec Redwood, Murdoch University) and assayed for relative IE1 expression as above.

### Macrophage transcriptomics

WT, *IfnαR* ^*-/-*^ and *Ifnβ1* ^*-/-*^ BM-DM were generated as previously described [60]. Cells were infected with WT-MCMV, MCMV_Δ_IE3 (MOI = 1) or were mock infected [75], and RNA isolated using RNeasy Mini kit at different time-points (Qiagen) according to manufacturer’s instructions. Some cells were stimulated with Poly(I:C) (Invivogen). Quality control (QC) was performed using an Agilent Bioanalyser and, total RNA labeled. RNA was hybridised to Mouse Gene 1.0ST microarrays (Affymetrix) using a WT Expression kit (Ambion, UK). GC metrics of captured data were assessed using Affymetrix Expression Console software and arrays were imported into Partek Genomics Suite (Partek) for downstream analysis. Arrays were normalised using the gcRMA algorithm [76] and data was filtered to include genes with at least 1 signal value of > = 150 across the time course.

### Ethics statement

All mice experiments were performed under the UK Home Office project Licence (PPL 30/2969). For human colon and PBMC samples, informed consent was obtained from participants in writing. The Wales Research Ethics Committee granted ethical approval for sample use in this study. For use of HCMV sero-positive donor PBMC samples used in this study informed consent was obtained in writing.

### Statistics

Power calculations were performed in R using data from a pilot study using CD4-Cre/IL-10 mice. It was determined that a minimum of 5 participants per group would be needed to detect a difference in means with 90% power and an alpha value set at 0.05. Statistical analysis was performed using the Mann-Whitney U test for paired analysis of viral-load analysis and flow cytometry data. Where more than 2 groups were assessed concurrently (viral-load analysis, ELISA analysis), 1-way ANOVA analysis of data was performed. To assess biological replication: all *in vivo* experiments were performed multiple times at different times, as stated in figure legends. All outliers were included in datasets, as shown. For all tests performed, p values are reported as *≤0.05, **≤0.01, and ***≤0.001.

## Supporting Information

S1 Fig CD4^+^ IL-10^+^ T cells are not T_H_17 or Tr1 cells. 10-BiT reporter mice were infected with MCMV and at day 7 and d14 pi spleen and salivary glands were isolated. Representative histograms of AhR viable (aqua live/dead^-^) Thy1.1^-^ or Thy1.1^+^ expression by CD4^+^CD3^+^ cells (top), control = fluorescent minus one-stained Thy1.1^+^ samples. Representative bivariant FACS plot of CD49b/LAG3 viable (aqua live/dead^-^) expression by CD4^+^CD3^+^ cells (bottom).
(TIFF)

S2 Fig Non-T cells do not produce compensatory IL-10 in CD4-Cre^+^IL-10^flox^ mice. CD4-Cre^-^IL-10^flox/flox^(Cre^-^) and CD4-Cre^+^IL-10^flox/flox^ (Cre^+^) mice were infected with MCMV and at day 7 and 14, IL-10 responses in the spleen and salivary glands were measured. Representative bivariant FACS plots of CD19^+^ and NK1.1^+^ versus IL-10 expression splenic (top) and salivary gland (d14 bottom) were analysed by viable (aqua live/dead^-^), CD19^+^/CD3^-^ and NK1.1^+^/CD3^-^ expression. Data is representative of 6 mice per group.
(TIFF)

S3 Fig Increased co-stimulatory ligand expression in the absence of T cell-derived IL-10 during MCMV infection. CD4-Cre^-^IL-10^flox/flox^(Cre^-^) and CD4-Cre^+^IL-10^flox/flox^ (Cre^+^) mice were infected with MCMV and at day 7 and 14, myeloid expression in the spleen (A) and salivary glands (B) was measured. CD11c^+^MHCII^+^ were quantified (left) and % CD80, CD86, OX-40L, 4-1BBL and CD40 expression (right) was assessed. Data is representative of 5–6 mice per group.
(TIFF)

S4 Fig MCMV does not induce autoimmunity in the absence of IL-10 production by CD4^+^ T-cells. (A) CD4-Cre^-^IL-10^flox/flox^ (Cre^-^) and CD4-Cre^+^IL-10^flox/flox^ (Cre^+^) mice were infected with MCMV and at day 14 and 30 spleen and salivary glands were isolated and CD4^+^/DR5^+^ cells were quantified. Mean ± SEM of total cells from 4–12 mice are shown and represent 2 separate experiments. (B) Cardiac punctures were performed d60 pi and anti-SSA IgG was measured by ELISA. Data is representative of 6 naïve and 16 mice in each group and is representative of 2 experiments.
(TIFF)

S5 Fig MCMV-specific memory CD8^+^ T-cell inflation is increased in the absence of IL-27R signaling. *Il-27r*α^-/-^ or WT mice were infected with MCMV and total virus-specific CD8^+^/IFNγ^+^ and CD8^+^/CD107a^+^ T-cells were quantified. Data is presented as mean ± SEM of 5 mice and is representative of 2 experiments.
(TIFF)

S6 Fig αICOS treatment does not impact on CD4 T_H_1 responses. WT (C57BL/6) mice were infected with MCMV and at d6 and d10 pi. 200 μg Isotype control or 200 μg αICOS was administered. At d14 pi splenic virus-specific CD4^+^/IFNγ^+^ responses were quantified and expressed at mean + SEM of 6 mice/group.
(TIFF)

S7 Fig Antagonizing of IL-10R and IL-27 during chronicity results in modest increase in virus-specific IFNγ^+^CD4^+^ T cells. WT (C57BL/6) mice were infected with MCMV treated with anti-IL-10R, anti-IL-27, and/or isotype on day 14 pi, and at d30 pi spleen CD4^+^/IFNγ^+^ responses were quantified and expressed as mean ± SEM of 4 mice/group.
(TIFF)
