# Supplementary figures and images for: Cytomegalovirus-Specific IL-10-Producing CD4+ T Cells Are Governed by Type-I IFN-Induced IL-27 and Promote Virus Persistence
Source: PLoS Pathog. 2016 Dec 7;12(12):e1006050. doi: 10.1371/journal.ppat.1006050 (PMC5142785; doi:10.1371/journal.ppat.1006050)

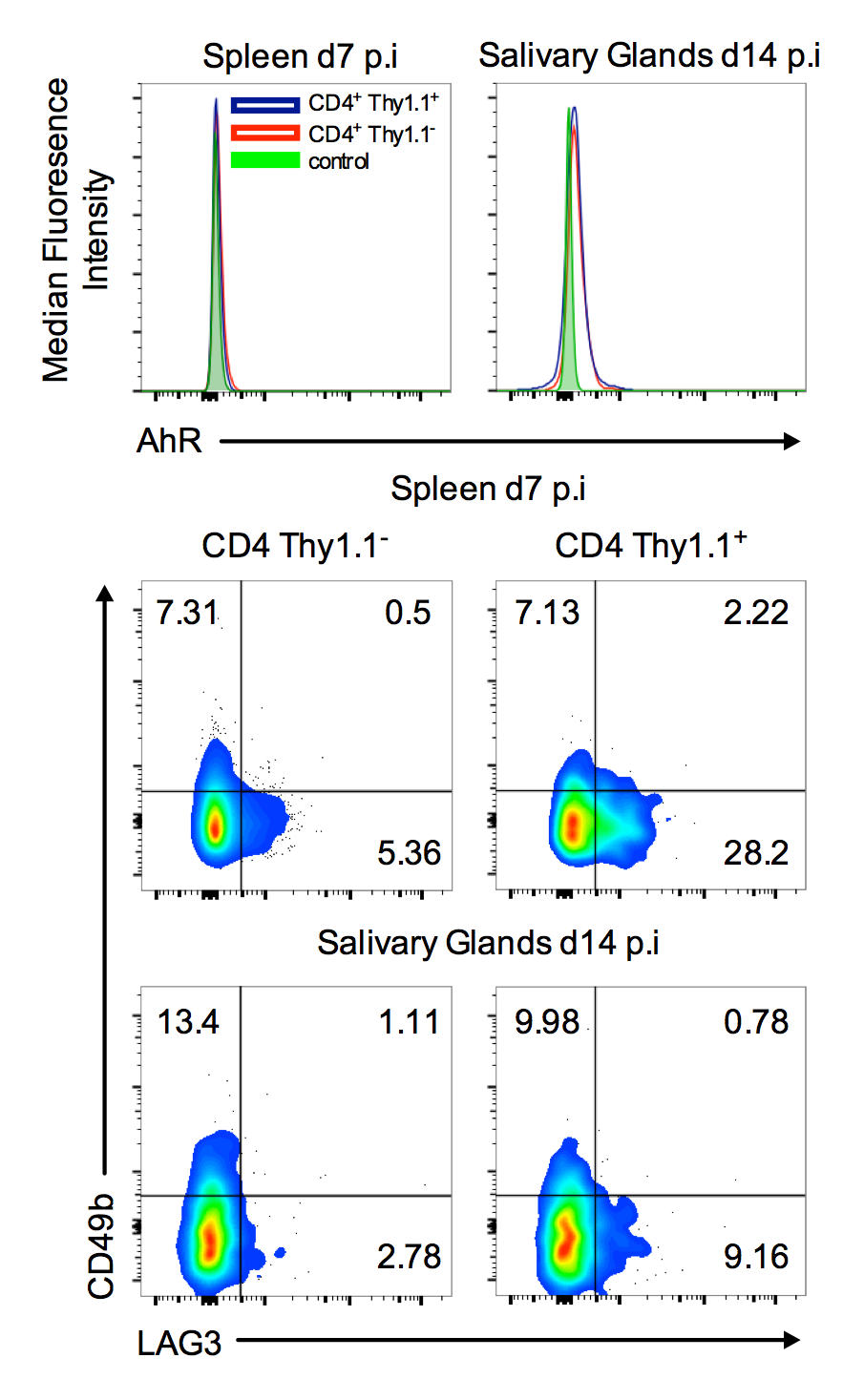

Supplement: S1 Fig — 10-BiT reporter mice were infected with MCMV and at day 7 and d14 pi spleen and salivary glands were isolated. Representative histograms of AhR viable (aqua live/dead-) Thy1.1- or Thy1.1+ expression by CD4+CD3+ cells (top), control = fluorescent minus one-stained Thy1.1+ samples. Representative bivariant FACS plot of CD49b/LAG3 viable (aqua live/dead-) expression by CD4+CD3+ cells (bottom). (TIFF) [file ppat.1006050.s001.tiff]

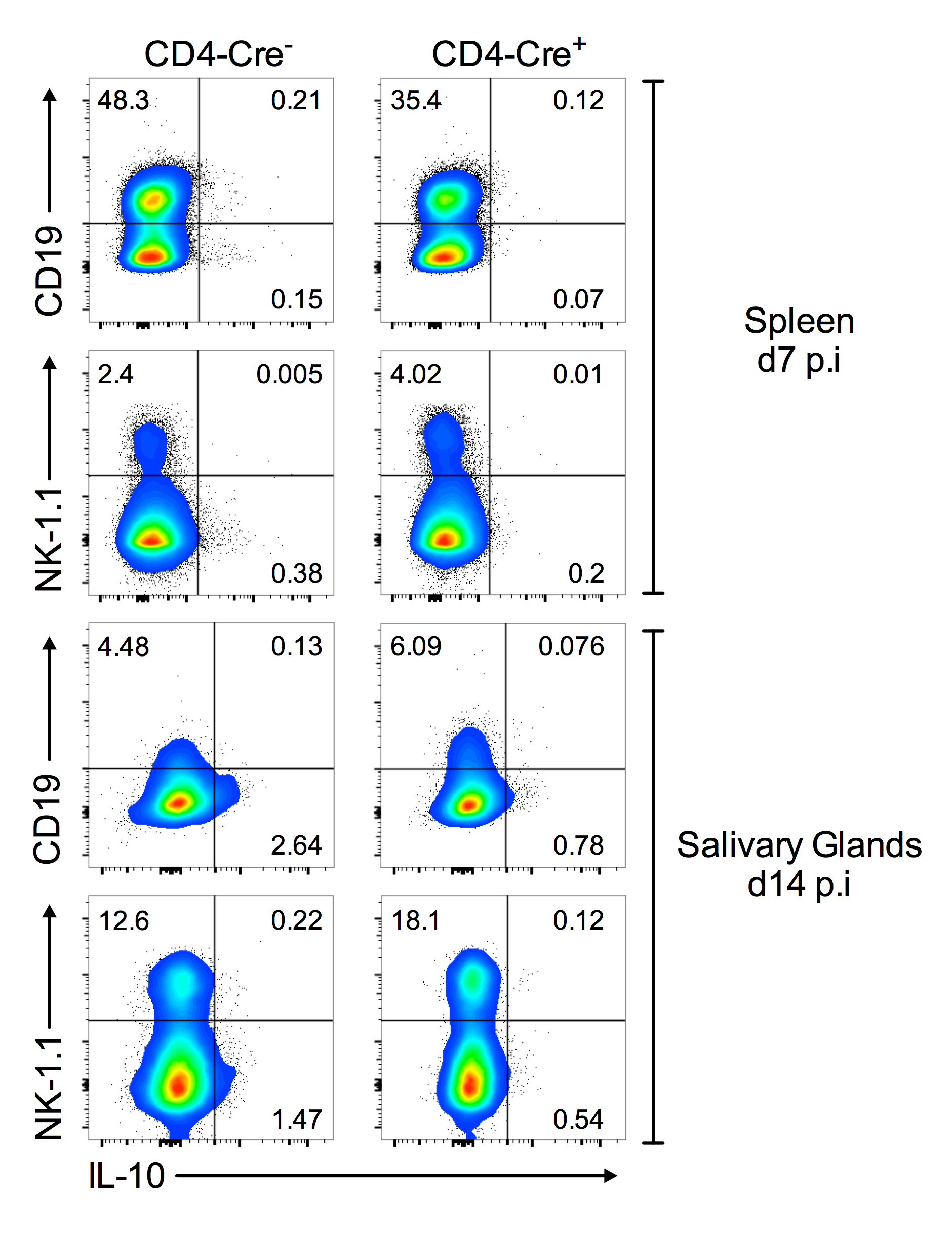

Supplement: S2 Fig — CD4-Cre-IL-10flox/flox(Cre-) and CD4-Cre+IL-10flox/flox (Cre+) mice were infected with MCMV and at day 7 and 14, IL-10 responses in the spleen and salivary glands were measured. Representative bivariant FACS plots of CD19+ and NK1.1+ versus IL-10 expression splenic (top) and salivary gland (d14 bottom) were analysed by viable (aqua live/dead-), CD19+/CD3- and NK1.1+/CD3- expression. Data is representative of 6 mice per group. (TIFF) [file ppat.1006050.s002.tiff]

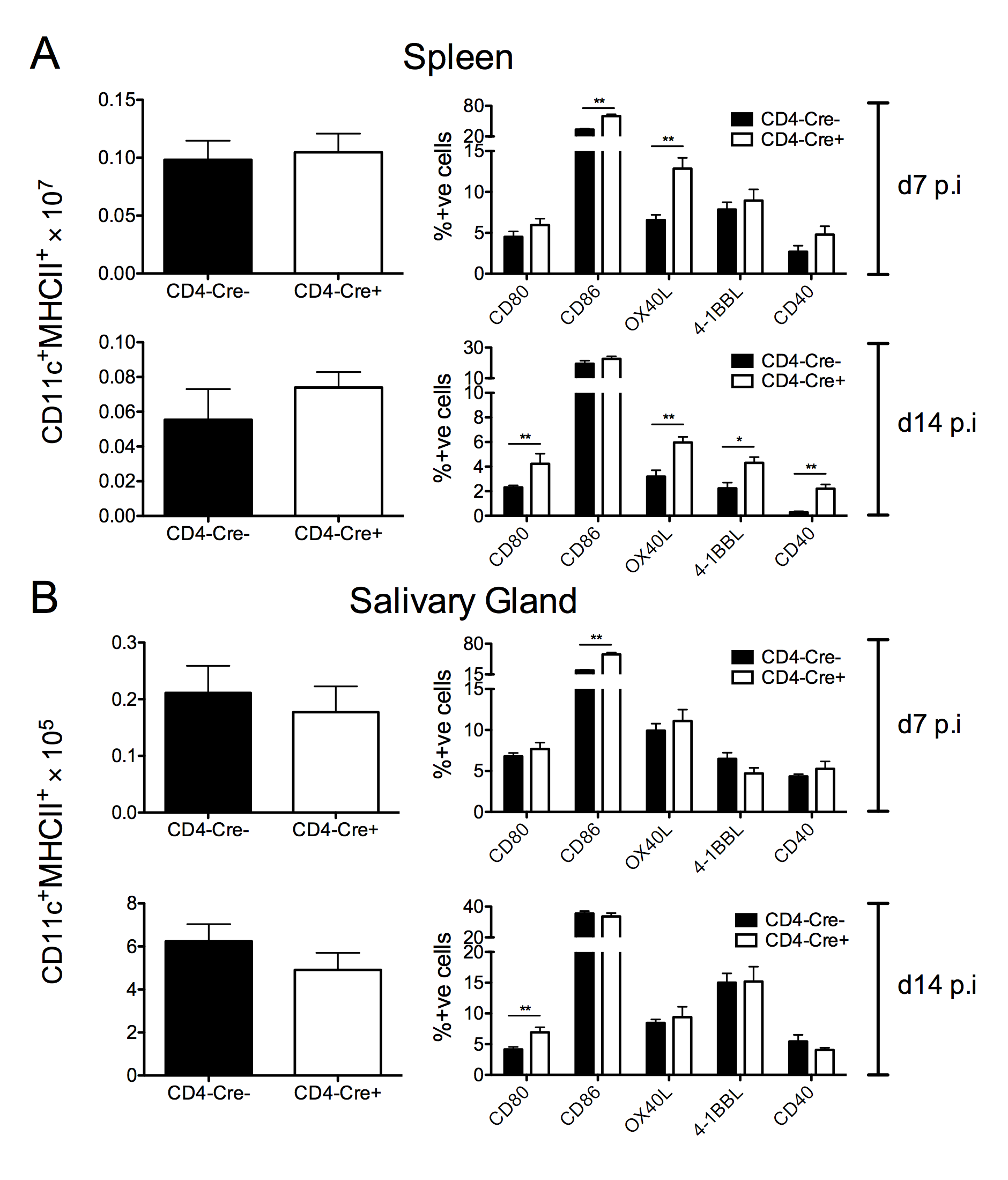

Supplement: S3 Fig — CD4-Cre-IL-10flox/flox(Cre-) and CD4-Cre+IL-10flox/flox (Cre+) mice were infected with MCMV and at day 7 and 14, myeloid expression in the spleen (A) and salivary glands (B) was measured. CD11c+MHCII+ were quantified (left) and % CD80, CD86, OX-40L, 4-1BBL and CD40 expression (right) was assessed. Data is representative of 5–6 mice per group. (TIFF) [file ppat.1006050.s003.tiff]

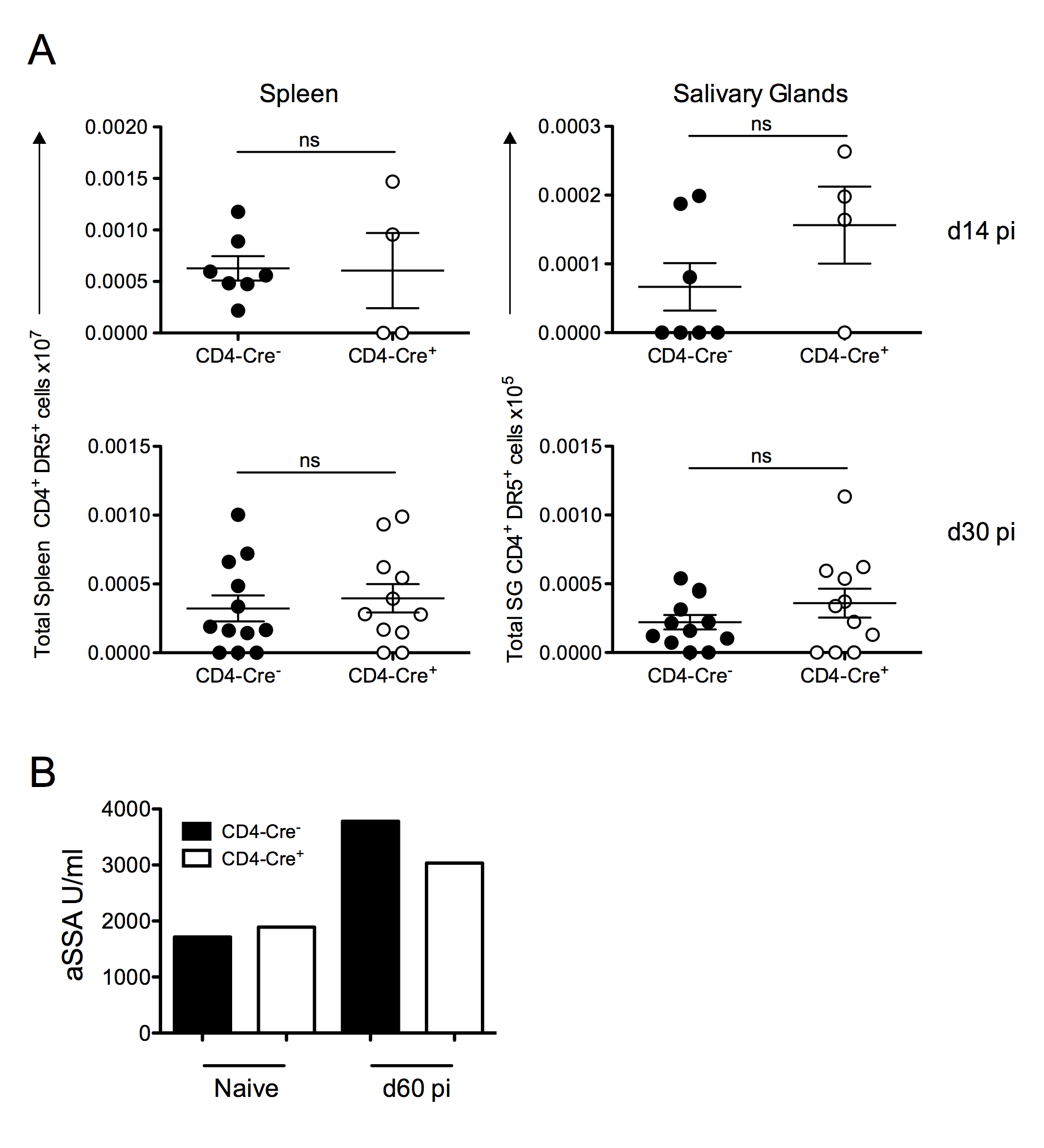

Supplement: S4 Fig — (A) CD4-Cre-IL-10flox/flox (Cre-) and CD4-Cre+IL-10flox/flox (Cre+) mice were infected with MCMV and at day 14 and 30 spleen and salivary glands were isolated and CD4+/DR5+ cells were quantified. Mean ± SEM of total cells from 4–12 mice are shown and represent 2 separate experiments. (B) Cardiac punctures were performed d60 pi and anti-SSA IgG was measured by ELISA. Data is representative of 6 naïve and 16 mice in each group and is representative of 2 experiments. (TIFF) [file ppat.1006050.s004.tiff]

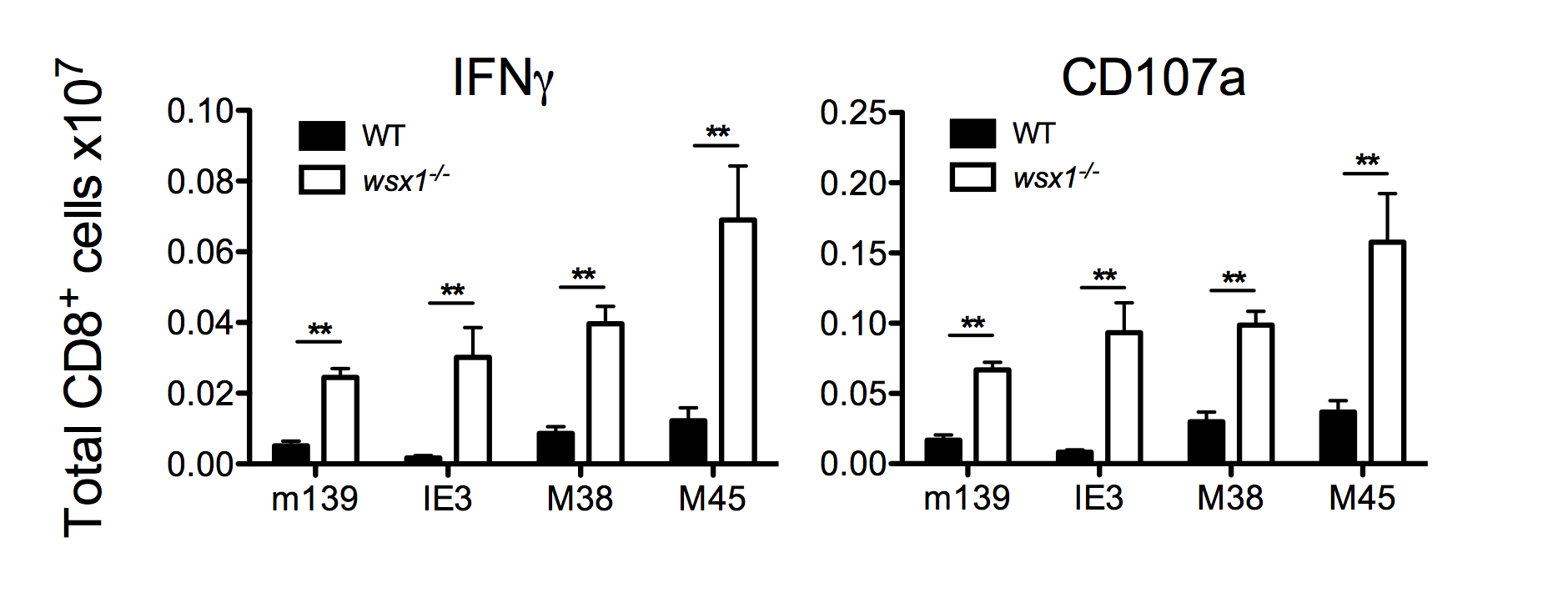

Supplement: S5 Fig — Il-27rα-/- or WT mice were infected with MCMV and total virus-specific CD8+/IFNγ+ and CD8+/CD107a+ T-cells were quantified. Data is presented as mean ± SEM of 5 mice and is representative of 2 experiments. (TIFF) [file ppat.1006050.s005.tiff]

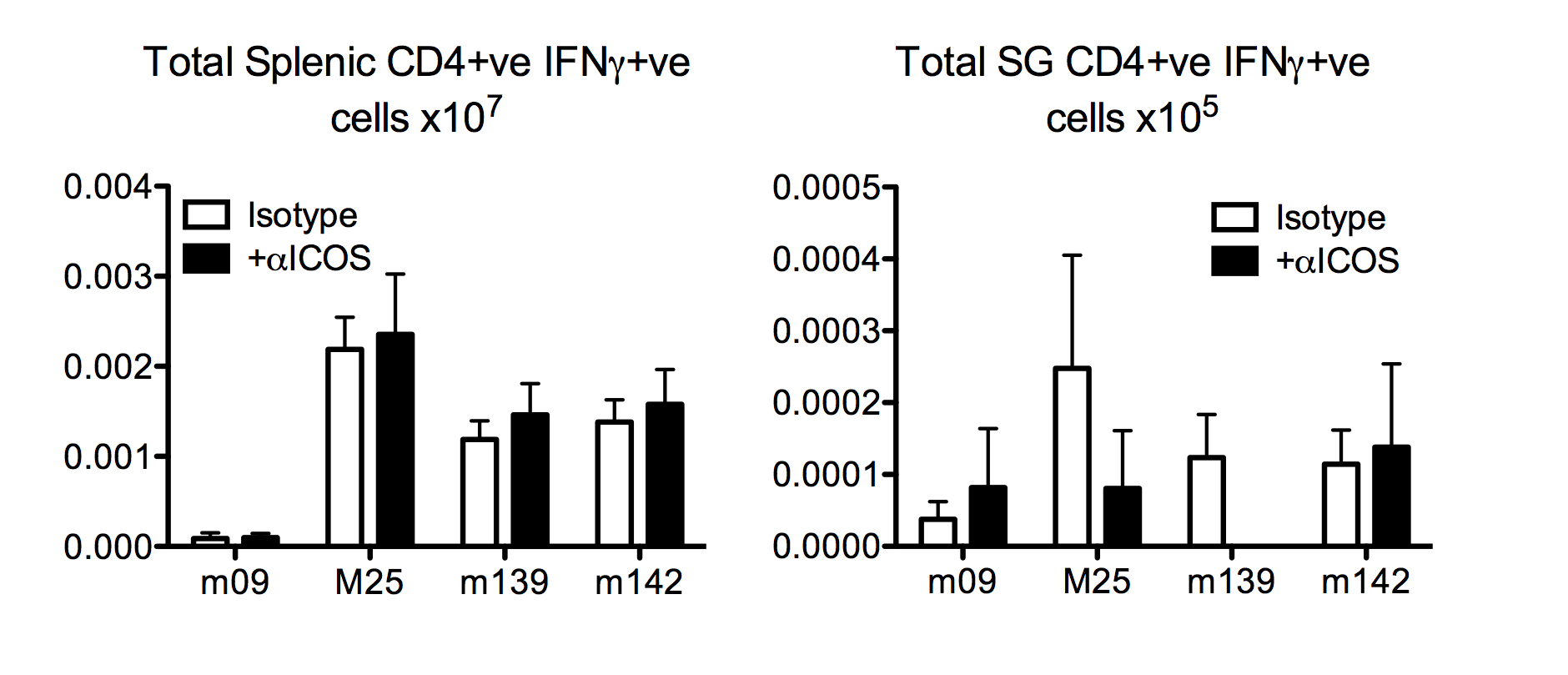

Supplement: S6 Fig — WT (C57BL/6) mice were infected with MCMV and at d6 and d10 pi. 200 μg Isotype control or 200 μg αICOS was administered. At d14 pi splenic virus-specific CD4+/IFNγ+ responses were quantified and expressed at mean + SEM of 6 mice/group. (TIFF) [file ppat.1006050.s006.tiff]

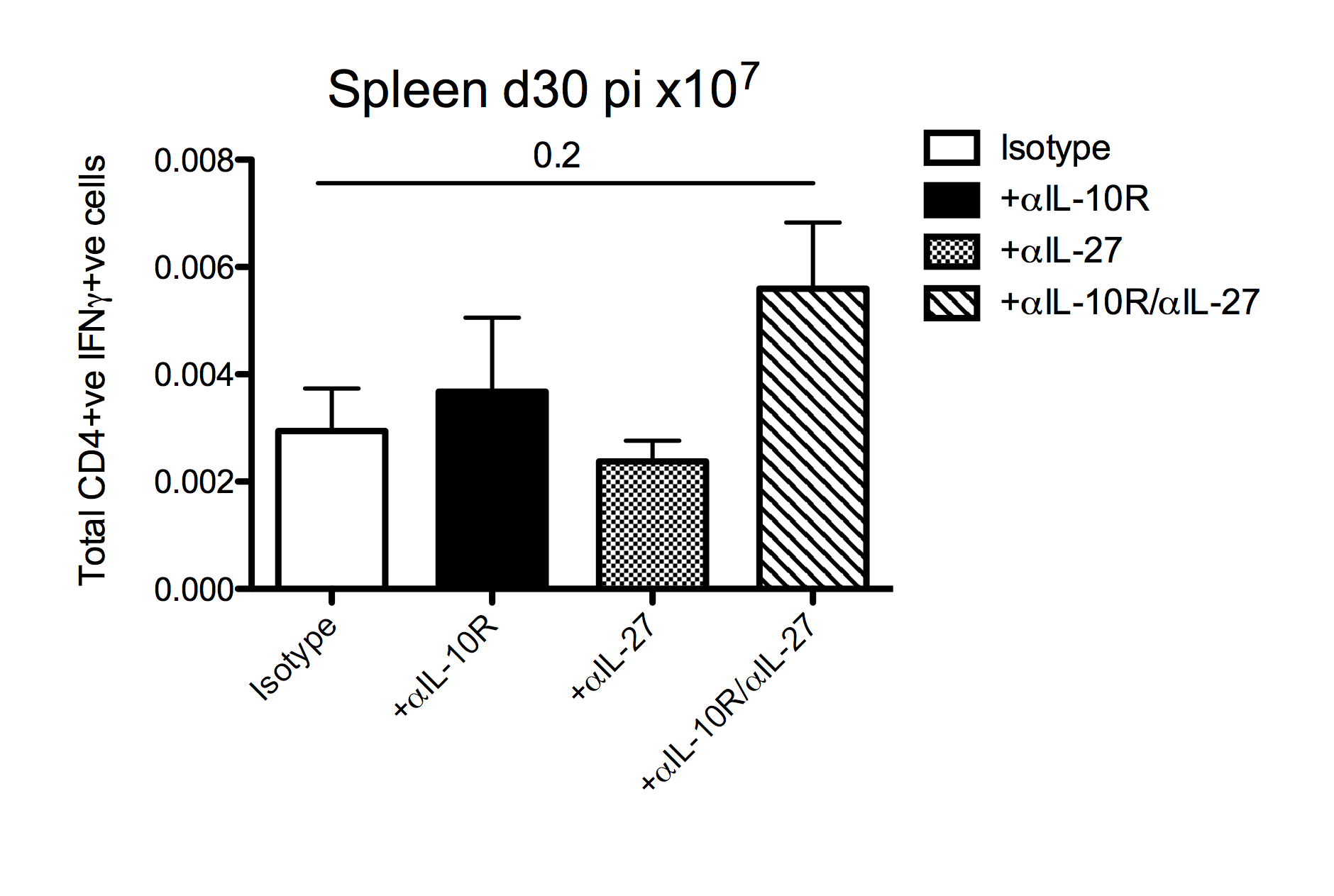

Supplement: S7 Fig — WT (C57BL/6) mice were infected with MCMV treated with anti-IL-10R, anti-IL-27, and/or isotype on day 14 pi, and at d30 pi spleen CD4+/IFNγ+ responses were quantified and expressed as mean ± SEM of 4 mice/group. (TIFF) [file ppat.1006050.s007.tiff]
